# Plasmonic Detection of SARS-CoV-2 Spike Protein with Polymer-Stabilized Glycosylated Gold Nanorods

**DOI:** 10.1021/acsmacrolett.1c00716

**Published:** 2022-02-20

**Authors:** Panagiotis G. Georgiou, Collette S. Guy, Muhammad Hasan, Ashfaq Ahmad, Sarah-Jane Richards, Alexander N. Baker, Neer V. Thakkar, Marc Walker, Sarojini Pandey, Neil R. Anderson, Dimitris Grammatopoulos, Matthew I. Gibson

**Affiliations:** † Department of Chemistry, University of Warwick, Gibbet Hill Road, CV4 7AL Coventry, U.K.; ‡ Warwick Medical School, University of Warwick, Gibbet Hill Road, CV4 7AL Coventry, U.K.; § Department of Physics, University of Warwick, Gibbet Hill Road, CV4 7AL Coventry, U.K.; ∥ Institute of Precision Diagnostics and Translational Medicine, University Hospitals Coventry and Warwickshire NHS Trust, Clifford Bridge Road Walsgrave, Coventry CV2 2DX, U.K.; ⊥ School of Life Sciences, University of Warwick, CV4 7AL Coventry, U.K.

## Abstract

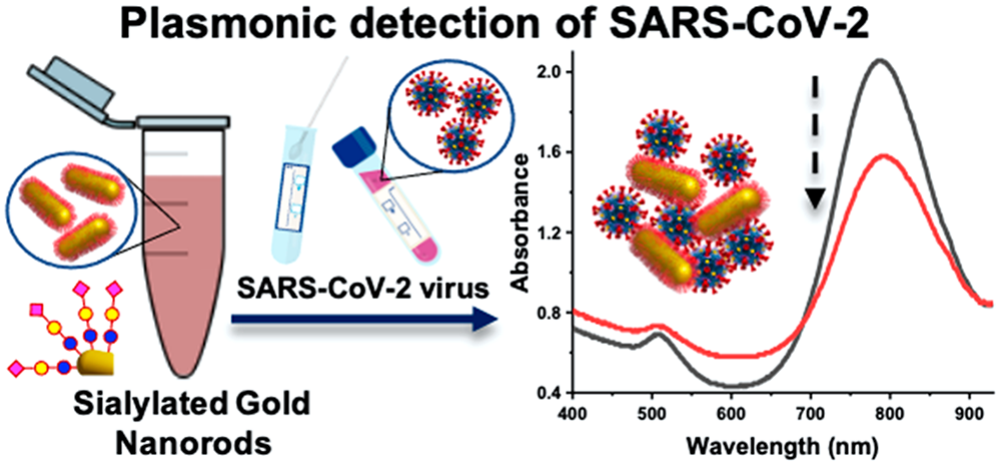

The COVID-19 pandemic has highlighted the need for innovative biosensing, diagnostic, and surveillance platforms. Here we report that glycosylated, polymer-stabilized, gold nanorods can bind the SARS-CoV-2 spike protein and show correlation to the presence of SARS-CoV-2 in primary COVID-19 clinical samples. Telechelic polymers were prepared by reversible addition–fragmentation chain-transfer polymerization, enabling the capture of 2,3-sialyllactose and immobilization onto gold nanorods. Control experiments with a panel of lectins and a galactosamine-terminated polymer confirmed the selective binding. The glycosylated rods were shown to give dose-dependent responses against recombinant truncated SARS-CoV-2 spike protein, and the responses were further correlated using primary patient swab samples. The essentiality of the anisotropic particles for reducing the background interference is demonstrated. This highlights the utility of polymer tethering of glycans for plasmonic biosensors of infection.

Current tools to detect viruses, including SARS-CoV-2 (viral agent responsible for COVID-19),^1^ incorporate molecular (genetic) tools such as reverse transcription–polymerase chain reaction (RT-PCR) that require trained personnel and infrastructure or cell-based assays.^2^ Lower-cost lateral flow immunoassay devices that use antibodies to detect and capture viral components have now emerged.^3^ However, there are other biological ligands that also interact with viruses that could also be exploited for biosensing, including glycans.^4^ Glycans dictate a large number of biological recognition processes including viral attachment, as exemplified by the strain-dependent binding of sialic acids by influenza hemeagglutinins.^5^ Many coronaviruses^6,7^ including Middle East respiratory syndrome (MERS)^8,9^ bind sialic acids, and we recently discovered that SARS-CoV-2 can bind *N*-acetyl neuraminic acid (NeuNAc).^10,11^ The putative binding site is located on the S1 domain of the spike protein.^10,12^ Whereas the biological role of sialic acid binding in SARS-CoV-2 is unclear,^13,14^ cell-surface sialic acids are known to promote infections of other coronaviruses;^9^ therefore, sialic acids show huge potential for use as ligands for SARS-CoV-2 capture. It is also notable that other glycan-binding functions of SARS-CoV-2 are also emerging,^15−17^ but there is much to understand about its glycobiology, and new tools to explore glycobiology are needed.

Gold (and other plasmonic) nanoparticles (AuNPs) have unique properties, such as high molar extinction coefficients, arising from LSPR (localized surface plasmon resonance). This makes them suitable scaffolds for a range of biosensing purposes,^18^ including lateral flow immunoassays,^19^ surface-enhanced Raman scattering (SERS),^20^ and aggregation-based assays where distance-dependent color changes (red–blue) occur due to SPR band coupling.^21^ Anisotropic AuNPs (rods, stars, cubes) are of particular interest in sensing applications.^22,23^ The position and intensity of their SPR peaks are more sensitive to perturbations in the local environment (such as binding events) than the isotropic equivalents.^24,25^ Gold nanorods (AuNRs) have two distinct SPR bands (transverse and longitudinal), unlike the single band found in spherical particles. Biological buffers or “real world” samples often have background coloration (such as from hemagglutinin in blood) that would obscure the ∼520 nm SPR band found in spherical gold but not the longitudinal band (>750 nm) in AuNRs. This makes AuNRs ideally suited for measurements in complex media or with primary patient samples.^26−29^ Furthermore, by functionalizing AuNRs with polymers, the colloidal stability of the system can be improved, decreasing the risk of false-positives while providing anchor sites for ligands such as glycans.^30,31^

We herein demonstrate plasmonic glycosylated AuNR biosensors for the detection of SARS-CoV-2. We use polymeric tethers to attach NeuNAc (and control glycans) onto AuNRs. The rods enable the plasmonic detection of a truncated SARS-CoV-2 spike protein from swab samples from COVID-19 patients. This demonstrates how polymer-tethered glycans can be used to introduce glycan functionality for infection biosensing and diagnostics with anisotropic particles.

To display the glycan, a telechelic poly(*N*-hydroxyethyl acrylamide) (PHEA) ligand was synthesized by RAFT (reversible addition–fragmentation chain-transfer) polymerization (Figure 1A), introducing a pentafluorophenyl (PFP) group at the ω-terminus (*M*_n,SEC_ = 7400 Da, *Đ*_M_ = 1.2) (Figures S1 and S2, Supporting Information (SI)). NeuNAc-α-2,3-Gal-β-1,4-Glc-GlycineNH_2_ (a commercially available amino-functional 2,3-sialyllactose derivative) was used to displace the PFP group, as confirmed by ^19^F nuclear magnetic resonance (NMR) and Fourier transform infrared (FTIR) analysis (Figures S5 and S6). This glycan was not optimized but was chosen to allow facile installation of the desired NeuNAc residue, as the terminal unit engages the spike protein.^12^ Galactosamine was also added to the chain end for use as a negative control. This polymeric glyco-ligand was immobilized onto AuNRs (10 × 38 nm, λ_MAX_ = 780 nm), and excess polymer was removed by multiple centrifugation/resuspension cycles. UV–vis spectroscopy revealed a red shift of the longitudinal LSPR band (Figure 1B) for the AuNRs, whereas dynamic light scattering (Figure 1C) confirmed the successful attachment of the glycopolymers to the particle surface, indicating multiple populations consistent with an anisotropic particle (bimodal size distribution corresponding to the AuNR diameter and length), and transmission electron microscopy (TEM) confirmed the particle structure (Figure 1D). The presence of the polymer was further confirmed by XPS (X-ray photoelectron spectroscopy) with samples containing polymer showing increased amine and amide peaks in the C 1s spectra and increased elemental ratios of N 1s versus Au 4f (Figures S7–S10 and Tables S1 and S2).

**Figure 1:**
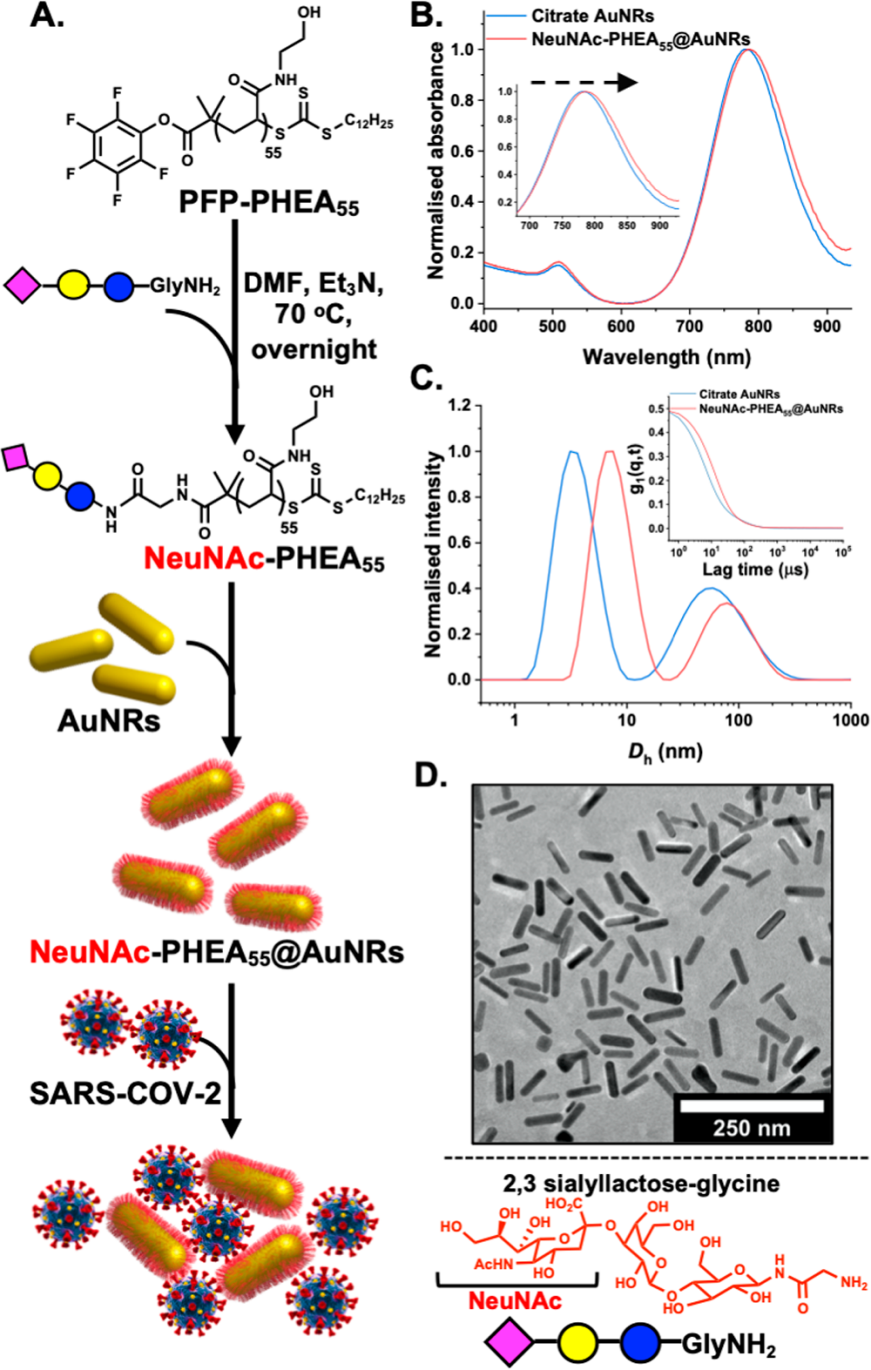
Glycosylated nanorods for SARS-CoV-2 detection. (A) Synthetic scheme for the synthesis of polymer-coated AuNRs bearing a NeuNAc terminal glycan and hypothesized interaction with SARS-CoV-2. (B) UV–vis spectra of AuNRs before/after polymer coating. (C) Dynamic light scattering of AuNRs before/after coating (inset: autocorrelation functions). (D) TEM image of NeuNAc-PHEA_55_@AuNRs.

A primary rationale for using rods rather than spherical gold for liquid biosensing is that the two distinct SPR bands enable monitoring of binding in the presence of interfering background components (due to spectral overlap).^28^ This is in contrast with spherical gold, which absorbs ∼520 nm and is subject to interference from media or serum components. The saline stability was assessed by a NaCl titration starting from 0.5 M (Figure S11) to eliminate any potential false-positives due to colloidal instability. It was observed NeuNAc-PHEA-coated particles remained stable for all saline concentrations.

Next, to evaluate the glycan-binding capacity, *Maackia amurensis* lectin II (MAL II) (with affinity for terminal α-2,3-linked sialic acids) was used as a positive control and soybean agglutinin (SBA) as a negative control (has affinity to β-GalNAc).^32,33^Figure 2A shows a strong dose-dependent response to MAL II with a strong decrease in absorbance at the LSPR_MAX_ and a shift in the LSPR wavelength consistent with nanorod binding responses (3.0–14.0 nm).^34^*Sambucus nigra* lectin (SNA), with preference for terminal α-2,6-linked sialic acids, was also tested. SNA lectin and its LSPR shift were compared with MAL II showing a smaller shift, as expected, due to the higher affinity for α-2,3-linked sialic acids (Figure S12). In contrast, SBA (Figure 2B) gave very small changes (1.0–7.0 nm), confirming that the NeuNAc residue can engage with lectins, but there are limited non-specific interactions. Non-specific interactions could be further reduced by an additional blocking step with bovine serum albumin (BSA) protein (Figures S14 and S15), but this was not essential for application. (Note: Studies were conducted for multiple batches of NeuNAcPHEA_55_@AuNRs.)

**Figure 2:**
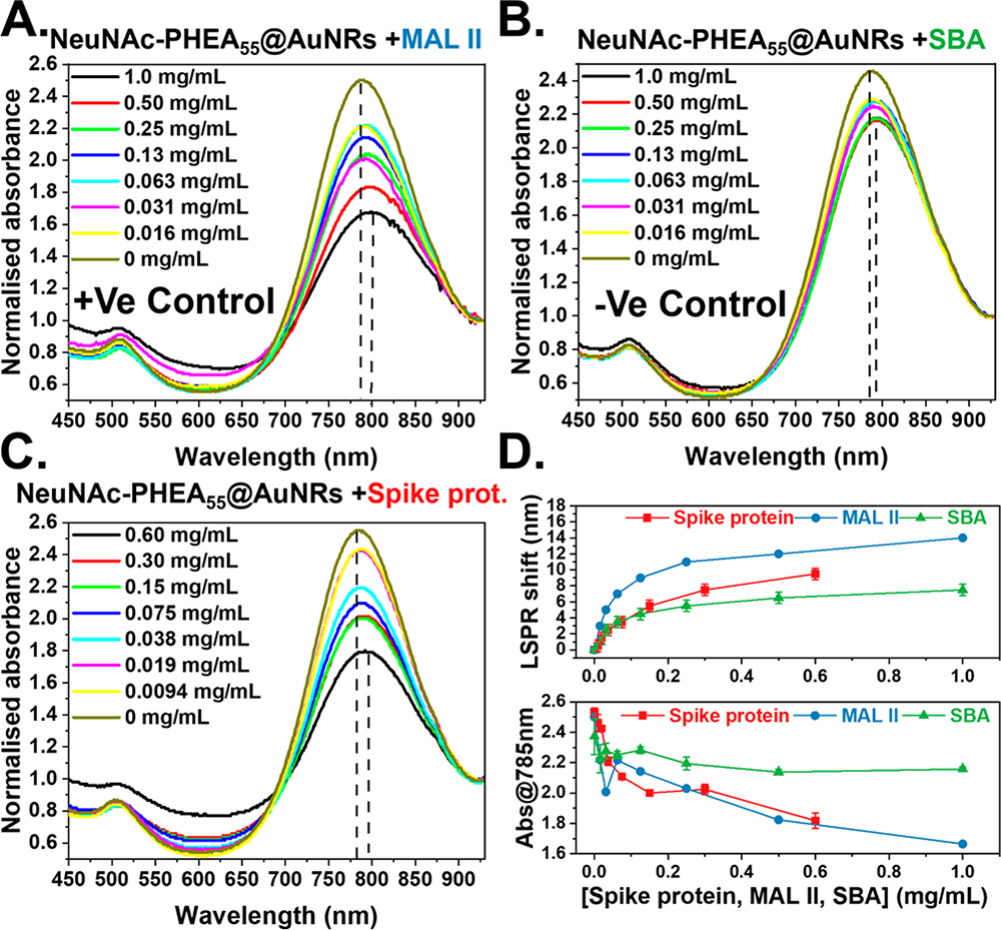
Lectin and spike protein binding responses of NeuNAc-PHEA_55_@AuNRs by UV–vis. (A) MAL II binding. (B) SBA binding. (C) SARS-CoV-2 spike protein binding. (D) Dose dependency of lectins on the LSPR and Abs_max_ (*N* = 3, mean ± SD).

To evaluate if the glycosylated AuNRs could recognize the spike protein, a truncated S1 domain of the spike protein (containing a single putative sialic acid binding site) was expressed in *E. coli* (Figures S3 and S4).^11^Figure 2C shows the strong dose-dependent response of the AuNRs to this truncated SARS-CoV-2 spike protein. There was a decrease in the absorbance at 785 nm (and a shift in the LSPR_max_, 1.0–10.0 nm), confirming that the glycosylated AuNRs are capable of engaging the spike protein and generating a signal response at 40 μg·mL^–1^. Figure 2D summarizes the spectra changes, showing that the change in Abs_785_ provides greater discrimination than the LSPR shift, although both features are expected to shift. To further confirm the selectivity, the galactosamine-functionalized rods were investigated, and limited spectral changes were observed when using the spike protein (LSPR shift <2.0 nm, Figure S13).

To highlight the importance of anisotropic particles (e.g., nanorods), spherical AuNPs (40 nm) were coated with NeuNAc-PHEA_55_ (Figure S17). Figure 3A shows how the absorption maxima for spherical particles overlap with the viral growth medium, whereas rods have no interference, which is a key benefit. The truncated spike protein used for the screening in Figure 2 has relatively low affinity on its own toward NeuNAc^10^ (the terminal glycan) and is monomeric (not the full-length protein). Figure 3B shows that spherical gold (which generates a signal due to aggregation) did not show significant spectral changes with this spike protein, confirming the second advantage of rods, which is that they can detect even when aggregation (due to monovalent targets or size-mismatch with analyte) does not occur. SARS-CoV-2 displays around 75 copies of each spike protein (25 trimers in electron microscopy^35^) and would benefit from multivalent enhancement. A SARS-CoV-2 spike protein expressing a pseudovirus (*Lentivirus*) was used to further probe the binding, but significant differences between positive and negative samples (Figures S18 and S19) were not clear, and hence primary samples were employed to directly probe the virus binding. (The SI has more details of this observation.)

**Figure 3:**
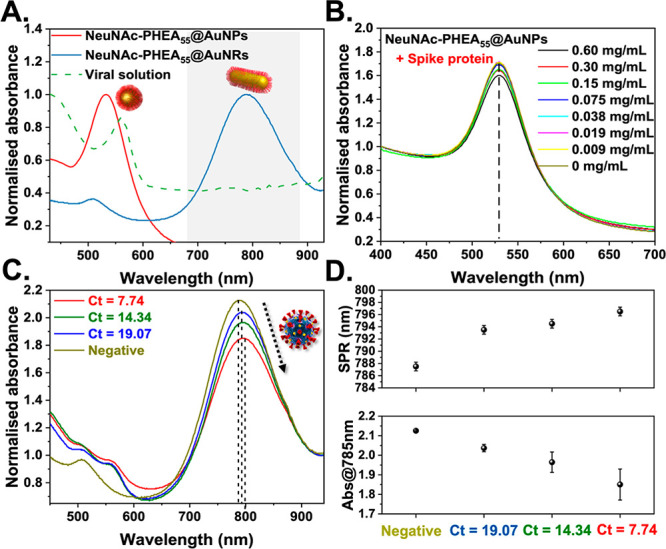
Nanorod-based detection of viral samples. (A) UV–vis spectra of AuNRs compared with spherical nanoparticles and virus media showing the optical window (gray shading). (B) UV–vis spectra of NeuNac-PHEA_55_@AuNP_40_ in the presence of SARS-CoV-2 spike protein. (C) UV–vis spectra of glycosylated AuNRs using clinical swab samples. (D) Summary of spectral changes for clinical swab samples (*N* = 3, mean ± SD). Ct = cycle threshold from RT-PCR. Negative = swab sample that tested negative by RT-PCR.

To show function in a complex sample matrix, a small panel of heat-inactivated primary clinical nasal swabs collected during the screening of suspected COVID-19 patients were evaluated. Samples had been tested by RT-PCR, generating a Ct value (threshold cycle) that was inversely proportional to the viral load (lower Ct numbers ∝ more virus). Samples with Ct values of 7.74, 14.34, and 19.07, and a negative sample, were incubated with the glyco-AuNRs and their UV–vis spectra were recorded for 1 h. (See the SI for time dependence; Figure S20A.) Figure 3D shows the LSPR shift and change in Abs_785nm_ for each sample. All positive samples show clear shifts in both LSPR and Abs_785nm_, whereas the negative sample gave no signal, confirming that the particles can engage the virus in primary clinical samples. Glycan binding for COVID-19 detection has been previously shown to correlate using flow-through devices.^11^ The stronger positives (lower Ct values) gave larger signals, showing, in principle, that this method can give viral load information. It was also clear in the spectra that background interference at ∼520 nm was present, demonstrating the advantage of particles with spectral features distinct from the background (Figure S20B). Whereas the format presented is not a ready-to-use diagnostic, it does demonstrate the principle that glycan binding can be used in complex primary samples to identify viral pathogens and may be useful in high-throughput setups for the initial screening of samples to focus on, for example, genetic screening. Fine-tuning of the glycan will be essential to introduce selectivity (over, e.g., influenza) as further structural details of the binding interaction emerge.^12,36^

In conclusion, we have demonstrated that polymer-tethered, NeuNAc-coated AuNRs can be used to detect the presence of SARS-CoV-2 spike protein using UV–vis spectroscopy. The glycan was incorporated onto the termini of polymeric tethers to introduce both viral targeting and colloidal stability (steric stabilization) to prevent false-positives that could occur due to medium- or saline-induced aggregation. Upon the addition of the recombinant spike protein, there was a dose-dependent response in the longitudinal (785 nm) absorption band. This is a key advantage of anisotropic AuNPs compared with spherical gold, as the radial plasmon band (∼520 nm) was subject to interference from the viral media. The signal output from the glycosylated nanorods using the primary clinical samples was in agreement with the Ct (cycle threshold) values from RT-PCR. Overall, this demonstrates that anisotropic plasmonic rods could be deployed in bioassays for viral detection, and because of the simple optical read-out, they may be well suited for automation or high-throughput applications. It is crucial to note that this is not a diagnostic but shows how glycan binding for sensing can be employed in complex liquid samples and offers a route to rapidly investigate new and emerging pathogens.

## Supporting Information Available

Full synthetic and characterization details, NaCl titration stability data, underpinning UV–vis traces, and lentiviral assay data and some additional discussion (PDF)
