# Supplementary material for: Plasmonic Detection of SARS-CoV-2 Spike Protein with Polymer-Stabilized Glycosylated Gold Nanorods
Source: ACS Macro Lett. 2022 Feb 20;11(3):317–22. doi: 10.1021/acsmacrolett.1c00716 (PMC8928465; doi:10.1021/acsmacrolett.1c00716)
Supplement: Supplementary file 1 — mz1c00716_si_001.pdf [file mz1c00716_si_001.pdf]

Supporting Information

"Plasmonic Detection of SARS-CoV-2 Spike Protein with Polymer-Stabilized Glycosylated Gold Nanorods"

Panagiotis G. Georgiou,[a] Collette S. Guy,[a,e] Muhammad Hasan,[a] Ashfaq Ahmad,[a] Sarah-Jane Richards,[a] Alexander N. Baker,[a] Neer V. Thakkar,[a] Marc Walker,[c] Sarojini Pandey,[d] Neil R. Anderson,[d] Dimitris Grammatopoulos,[b,d] and Matthew I. Gibson[*a,b]*

[a] Department of Chemistry, University of Warwick, Gibbet Hill Road, CV4 7AL, Coventry, UK

[b] Warwick Medical School, University of Warwick, Gibbet Hill Road, CV4 7AL, Coventry, UK

[c] Department of Physics, University of Warwick, Gibbet Hill Road, CV4 7AL, Coventry, UK

[d] Institute of Precision Diagnostics and Translational Medicine, University Hospitals Coventry and Warwickshire NHS Trust, Clifford Bridge Road Walsgrave, Coventry, CV2 2DX

[e] School of Life Sciences, University of Warwick, UK, CV4 7AL

*<u>*Corresponding Author*</u>: m.i.gibson@warwick.ac.uk (M.I.G.)

Contents

Experimental Section S3

Materials and Methods S3

Materials S3

Characterization Techniques S4

Synthetic Section S6

Methods S8

Supporting Characterization Data for PFP/NeuNac-PHEA$_{55}$ S13

Supporting X-Ray Photoelectron Spectroscopy Data for PFP/NeuNac-PHEA$_{55}$-functionalized AuNRs S14

Supporting Characterization Data for Colloidal Stability and Spike protein/Lectin-Binding Studies of NeuNAc/Gal-PHEA$_{55}$-Functionalized AuNRs 139

Supporting Characterization Data of NeuNAc-PHEA$_{55}$-Functionalized AuNRs in Response to Lentiviral and Clinical Swab Samples 13

References S28

Experimental Section

Materials and Methods

Materials

All chemicals were used as supplied unless otherwise stated. Citrate-stabilized gold nanorods (GNRs) of 10 nm width and 38 nm length (λ_{max} = 780 nm, OD = 1) were purchased from Nanopartz. 2-(Dodecylthiocarbonothioylthio)-2-methylpropionic acid pentafluorophenyl ester (98%, PFP-DMP), *N*-hydroxyethyl acrylamide (97%, HEA), triethylamine (>99%), HEPES (≥99.5%), PBS and bovine serum albumin (≥98%, BSA) were purchased from Sigma-Aldrich. Citrate stabilized gold nanoparticles (AuNPs, OD = 1) of 40 nm diameter were also purchased from Sigma-Aldrich. Sodium chloride (≥99.5%) and calcium chloride were purchased from Thermo Fisher Scientific. NeuNAc-α(2,3)-Gal-β-1,4-Glc-GlycineNH_2 (>95%) was obtained from Carbosynth. Soybean agglutinin (SBA), *Maackia amurensis* lectin II (MAL II) and *Sambucus nigra* (SNA) lectins were purchased from Vector Laboratories. D(+)-galactosamine hydrochloride (99%) was purchased from Acros Organics.

Spike (SARS-CoV-2) pseudotyped lentivirus (Luc Reporter) (Catalogue number: 79942, Lot number: 200730) and bald lentiviral pseudovirion (Luc reporter) (Catalogue number: 79943, Lot number: 200727) were purchased from AMSBIO. Clear and black half area 96-well plates were purchased from Greiner Bio-one. Formvar coated copper grids were purchased from EM Resolutions. Distilled water used for buffers was MilliQ grade >18.2 mΩ resistance.

Swab Samples:

This study used remnant elutions from nasal, or nasal + oral swab samples collected from symptomatic staff/patients at the University Hospital Coventry and Warwickshire NHS Trust and routinely tested by standard PCR protocols employing the Abbott assay (Ref: 09N77-095, https://www.molecular.abbott/sal/9N77-095_SARS-CoV-2_US_EUA_Amp_PI.pdf) during April-September 2020.[1] As this evaluation study used left-over anonymized material no written informed consent was obtained, although the project was registered with the local COVID-19 research committee. Dry cotton swabs, one nose and one throat, were obtained in a single universal container. To each primary swab sample was added 2000 µL of molecular grade water (if one swab) or 2500 µL of molecular grade water (if two swabs are in universal container). These were then vortexed and allowed to settle for 5 minutes. All liquid was transferred from primary container into 13 mm × 75 mm tube. These tubes are heat inactivated

at 85 °C for 10 minutes. The specimens were then used for testing. All testing was conducted on samples which had been frozen at -80 °C.

Characterization Techniques

NMR Spectroscopy. ^{1}H-NMR, ^{13}C-NMR and ^{19}F-NMR spectra were recorded at 300 MHz or 400 MHz on a Bruker DPX-300 or DPX-400 spectrometer respectively, with methanol-d_4 as the solvent. Chemical shifts of protons are reported as δ in parts per million (ppm) and are relative to solvent residual peak (CH_3OH, δ = 3.31 ppm).

FT-IR Spectroscopy. Fourier Transform-Infrared (FT-IR) spectroscopy measurements were carried out using an Agilent Cary 630 FT-IR spectrometer, in the range of 650 to 4000 cm^{-1}.

Size Exclusion Chromatography. Size exclusion chromatography (SEC) analysis was performed on an Agilent Infinity II MDS instrument equipped with differential refractive index (DRI), viscometry (VS), dual angle light scatter (LS) and variable wavelength UV detectors. The system was equipped with 2 x PLgel Mixed D columns (300 x 7.5 mm) and a PLgel 5 µm guard column. The mobile phase used was DMF (HPLC grade) containing 5 mM NH_4BF_4 at 50 °C at flow rate of 1.0 $mL.min^{-1}$. Poly(methyl methacrylate) (PMMA) standards (Agilent EasyVials) were used for calibration between 955,000 – 550 $g.mol^{-1}$. Analyte samples were filtered through a nylon membrane with 0.22 μm pore size before injection. Number average molecular weights (M_n), weight average molecular weights (M_w) and dispersities ($Đ_M$ = M_w/M_n) were determined by conventional calibration and universal calibration using Agilent GPC/SEC software.

Dynamic Light Scattering. Hydrodynamic diameters (D_h) and size distributions of particles were determined by dynamic light scattering (DLS) using a Malvern Zetasizer Nano ZS with a 4 mW He-Ne 633 nm laser module operating at 25 °C. Measurements were carried out at an angle of 173° (back scattering), and results were analyzed using Malvern DTS 7.03 software. All determinations were repeated 5 times with at least 10 measurements recorded for each run. D_h values were calculated using the Stokes-Einstein equation where particles are assumed to be spherical.

UV-Vis Spectroscopy. Absorbance measurements BioTek Epoch microplate reader in the wavelength (λ) range of 400-990 nm (step = 1 nm).

Transmission Electron Microscopy. Dry-state stained TEM imaging was performed on a JEOL JEM-2100Plus microscope operating at an acceleration voltage of 200 kV. All dry-state samples were diluted with deionized water and then deposited onto formvar-coated copper grids.

X-ray Photoelectron Spectroscopy. The X-ray photoelectron spectroscopy (XPS) data were collected at the Warwick Photoemission Facility, University of Warwick. The samples were attached to electrically-conductive carbon tape, mounted on to a sample bar and loaded in to a Kratos Axis Ultra DLD spectrometer which possesses a base pressure below 1 x 10^{-10} mbar. XPS measurements were performed in the main analysis chamber, with the sample being illuminated using a monochromated Al Kα x-ray source. The measurements were conducted at room temperature and at a take-off angle of 90° with respect to the surface parallel. The core level spectra were recorded using a pass energy of 20 eV (resolution approx. 0.4 eV), from an analysis area of 300 μm x 700 μm. The spectrometer work function and binding energy scale of the spectrometer were calibrated using the Fermi edge and $3d_{5/2}$ peak recorded from a polycrystalline Ag sample prior to the commencement of the experiments. In order to prevent surface charging the surface was flooded with a beam of low energy electrons throughout the experiment and this necessitated recalibration of the binding energy scale. To achieve this, the C-C/C-H component of the C 1*s* spectrum was referenced to 285.0 eV. The data were analyzed in the CasaXPS package, using Shirley backgrounds and mixed Gaussian-Lorentzian (Voigt) lineshapes. For compositional analysis, the analyser transmission function has been determined using clean metallic foils to determine the detection efficiency across the full binding energy range.

Circular Dichroism. Circular dichroism (CD) analysis of truncated SARS-CoV-2 Spike (S1) protein (first 300 amino acids) was performed on a Jasco J-1500 CD spectropolarimeter, featuring a 150 W air-cooled Xe lamp and a Jasco Peltier PTC-423S/15 temperature controlling system. The sample was prepared at 0.2 mg/mL concentration, in 10mM sodium phosphate buffer (pH = 7.4) and contained in a 1mm pathlength quartz cuvette (Hellma, USA) with a spectral bandwidth of 1.0 nm. All spectra obtained were the mean of six independent scans post calibration with sodium phosphate buffer. Data collected were analyzed and processed with the online tool “DICHROWEB”.

Synthetic Section

Polymerization of *N*-(2-hydroxyethyl)acrylamide (HEA) using 2-(dodecylthiocarbonothioylthio)-2-methylpropanoic acid pentafluorophenyl ester (PFP-DMP) chain transfer agent (CTA).

A vial was charged with 2-(dodecylthiocarbonothioylthio)-2-methylpropanoic acid pentafluorophenyl ester (PFP-DMP) (100 mg, 0.19 mmol, 1 eq), *N*-(2-hydroxyethyl) acrylamide (HEA) (1.30 g, 11.3 mmol, 60 eq), AIBN (3.1 mg, 0.02 mmol, 0.1 eq), and 7 mL of dioxane/methanol mixture (1:1). The vial was then sealed and deoxygenated using three successive cycles of freeze-pump-thaw to remove O_2(g). The vial was placed into an aluminium heating block which had been pre-heated to 70 °C to initiate polymerization. After 2 h, the polymerization was quenched by exposing the vial to air and submerging it into liquid N_2. An aliquot was withdrawn for determination of monomer conversion by ^{1}H NMR spectroscopy. The polymer was precipitated into diethyl ether from dioxane twice to yield a yellow polymer product that was further dried under vacuum. Conversions were calculated using ^{1}H NMR spectroscopy by comparing the integrations of the HEA monomer signals (δ 5.67 ppm) with those of the corresponding signals of the polymer (δ 2.22–2.04 ppm, CH of PHEA backbone). $M_{n,NMR}$ was calculated by end-group analysis by comparing the integrations of the –CH_3 signals (δ 0.92 ppm) of dodecyl end-group with those of the corresponding signals of the polymer (δ 2.22–2.04 ppm).^{1}H NMR (400 MHz, CD_3OD): δ (ppm) 8.15–8.03 (br m, N***H*** of PHEA side chain), 3.89–3.13 (br m, NH–C***H₂*** and C***H₂***–OH of PHEA side chain), 2.35–2.05 (br m, C***H*** of PHEA backbone), 1.85–1.31 (br m, C***H₂*** of PHEA backbone), 0.92 (t, 3H, CH_2–C***H₃*** of dodecyl end-group). $M_{n,NMR}$ = 6800 g mol^{-1} ($DP_{PHEA,\ NMR}$ = 55). SEC (5 mM NH_4BF_4 in DMF) $M_{n,\ SEC\ RI}$ = 7400 g.mol^{-1}, $Đ_{M,\ SEC\ RI}$ = 1.24. FT-IR (neat): ν (cm^{-1}) 3300 (N–H and O–H stretch); 2868 (alkyl C–H stretch); 1772 (C_6F_5C=O stretch); 1638(amide C=O stretch); 1544 (N–H bend); 1438 (alkane); 1216 (C–O stretch); 1060 (C–O stretch); 950 (C–F peak on shoulder of 1060 peak).

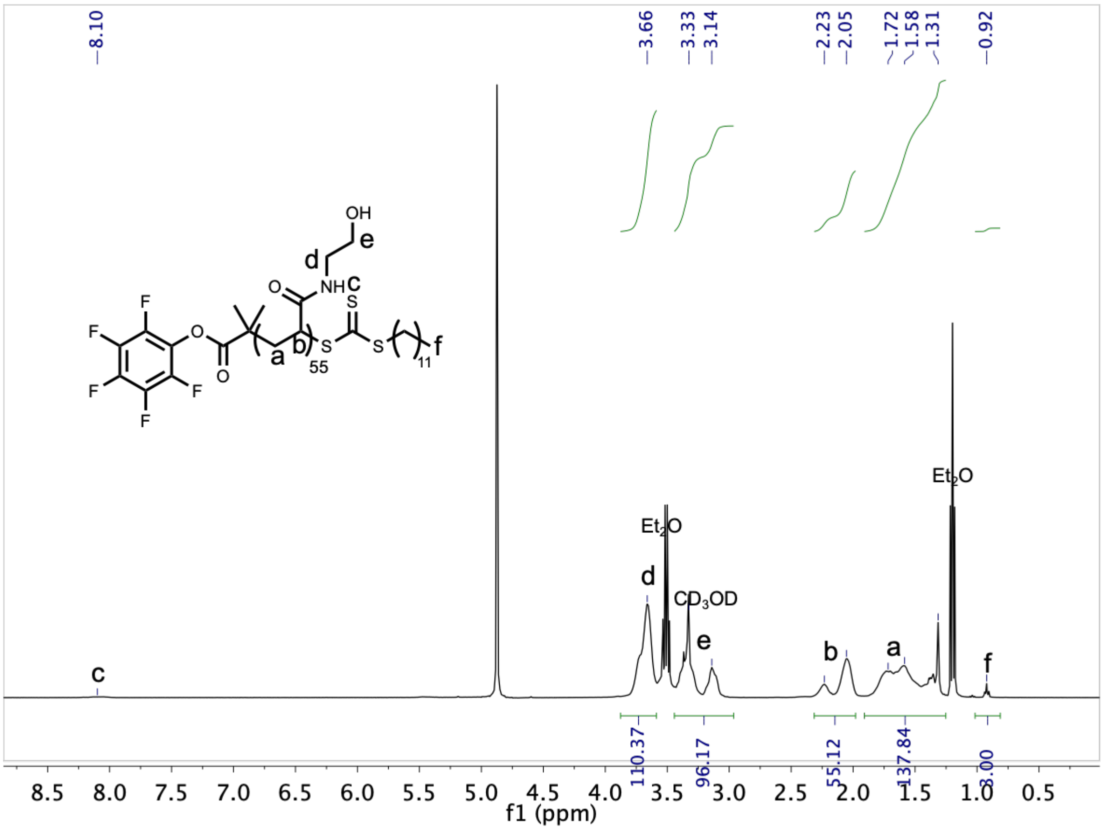

Figure S1. ^{1}H-NMR spectrum of PFP-PHEA$_{55}$ homopolymer recorded in methanol-d_4.

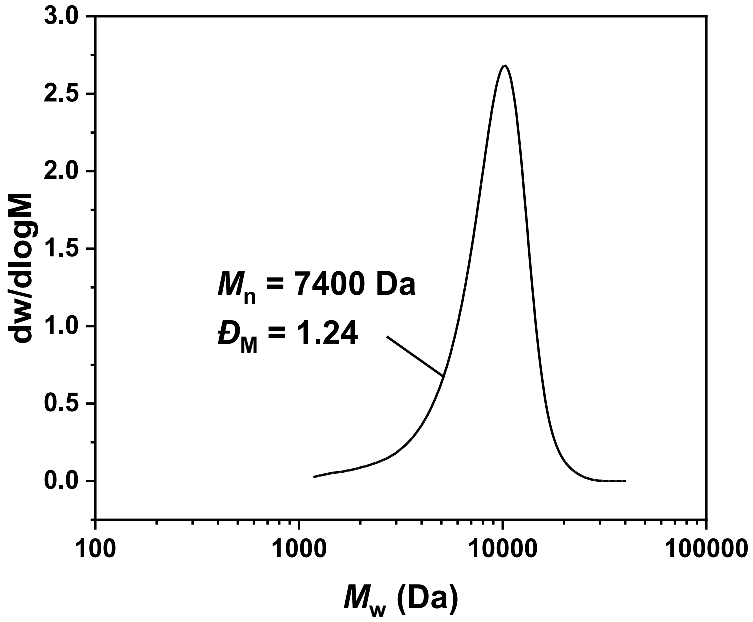

Figure S2. Size exclusion chromatography analysis. Normalized SEC RI molecular weight distribution for PFP-PHEA$_{55}$ homopolymer. M_n and $Đ_M$ values were calculated from PMMA standards using 5 mM NH_4BF_4 in DMF as the eluent.

Methods

End-group modification of PFP-poly(N-hydroxyethyl acrylamide) (PFP-PHEA) homopolymers using NeuNAc-α(2,3)-Gal-β-1,4-Glc-GlycineNH$_2$ and galactosamine hydrochloride. In a typical reaction, PFP-PHPMA$_{55}$ (11 mg, 0.0015 mmol, 1 eq), NeuNAc-*α*-2,3-Gal-*β*-1,4-Glc-GlycineNH$_2$ (2 mg, 0.003 mmol, 2 eq) or galactosamine hydrochloride (0.7 mg, 0.003 mmol, 2 eq) were dissolved in 1 mL DMF with 0.05 M triethylamine (TEA) (5 µL). The reaction was stirred at 50 °C for 16 hrs. The polymer was precipitated into diethyl ether from methanol three times and dried under vacuum. ^{19}F-NMR and FT-IR analyses indicated loss of the pentafluorophenyl (PFP) ester.

Gold nanorod functionalization. Approximately 1 mg of the desired thiol-terminated glycosylated homopolymer (NeuNAc-PHEA$_{55}$/ Gal-PHEA$_{55}$) was added to a micro-centrifuge tube and dissolved in 100 µL of MiliQ water. 900 µL of the citrate-stabilized gold nanorod solution (OD = 1) was added to this that was then agitated for 1 hour in the absence of light. To remove excess polymer, nanorods were centrifuged and following careful removal of the supernatant were then redispersed in 1 mL of MilliQ water and the centrifugation-resuspension process repeated for a total of three cycles. After the final cycle the nanorods were dispersed in 1 mL of MilliQ water for future use. TEM, DLS and UV-Vis analyses were performed on the sample after dilution to an appropriate analysis concentration.

BSA blocking of nanoparticle surface. 1 mL of NeuNAc-PHEA$_{55}$@AuNRs (OD = 1) were centrifuged at 10,00 rpm for 10 mins and aqueous supernatant was removed and replaced with 1 mL of 1 mg/mL BSA for 30 minutes. Excess of BSA was then removed by three centrifugation/ redispersion cycles. Resulted nanoparticle solution was characterized by DLS.

Protein Expression

Recombinant expression and purification of truncated SARS-CoV-2 Spike (S1) protein (first 300 amino acids). A pET21a plasmid encoding for a hexahistidine-tag, SUMO-tag and the first 300 amino acids of SARS-CoV-2 was purchased from Genscript Inc. The plasmid was transformed into competent Escherichia coli BL21(DE3) cells (New England Biolabs). A colony was selected to inoculate 100 mL of LB-medium containing 100 µg.mL-1 kanamycin and was grown overnight at 37 °C under continuous shaking of 180 rpm. The following day,

10 mL of the preculture was added to 1 L of LB-medium (supplemented with 100 µg.mL-1 kanamycin) in a 2.5 L Ultra Yield™ flask and grown at 37 °C with a shaking speed of 180 rpm till on OD600 of 0.6 was reached. The temperature was then reduced to 16 °C and the cells incubated for another hour before adding IPTG (isopropyl *β*-D-1-thiogalactopyranoside) to a final concentration of 0.2 mM. The overexpression of the protein was allowed to take place overnight following which the cells were centrifuged at 5000 g for 10 minutes at 4 °C. Pelleted cells were resuspended in PBS supplemented with Pierce protease inhibitor mini-tablets. The suspension was passed through a STANSTED 'Pressure Cell' FPG12800 homogenizer in order to lyse the cells. The cell lysate was centrifuged at 48,000 g and the supernatant was passed through a 0.45 µm filter before being added to a 3 mL column of IMAC cOmplete His-Tag Purification Resin (Roche) pre-equilibrated with PBS. The column was washed with 20 column volumes of PBS. Bound protein was eluted using 6 mL of 300 mM Imidazole in PBS. Further purification of was achieved using a HiLoad 16/600 Superdex 200 pg gel filtration column (GE Healthcare) with PBS as the running buffer. Purity was estimated using SDS-PAGE and protein concentration determined using Thermo Scientific Pierce BCA assay kit. Various volumes of the protein contained in PBS solution were aliquoted into 1.5 mL microcentrifuge tubes and snap-frozen in liquid nitrogen to store at -80 °C till required. Protein sequence expressed (N-terminal polyhistidine and SUMO tags with the first 300 amino acids of the spike protein);
MGSSHHHHHHGSGMSDSEVNQEAKPEVKPEVKPETHINLKVSDGSSEIFFKIKKTTPL
RRLMEAFAKRQGKEMDSLRFLYDGIRIQADQTPEDLDMEDNDIIEAHREQIGGGSEF
ELMFVFLVLLPLVSSQCVNLTTRTQLPPAYTNSFTRGVYYPDKVFRSSVLHSTQDLFL
PFFSNVTWFHAIHVSGTNGTKRFDNPVLPFNDGVYFASTEKSNIIRGWIFGTTLDSKT
QSLLIVNNATNVVIKVCEFQFCNDPFLGVYYHKNNKSWMESEFRVYSSANNCTFEY
VSQPFLMDLEGKQGNFKNLREFVFKNIDGYFKIYSKHTPINLVRDLPQGFSALEPLVD
LPIGINITRFQTLLALHRSYLTPGDSSSGWTAGAAAYYVGYLQPRTFLLKYNENGTIT
DAVDCALDPLSETK (NB: The 300 amino acids of the spike protein are underlined)

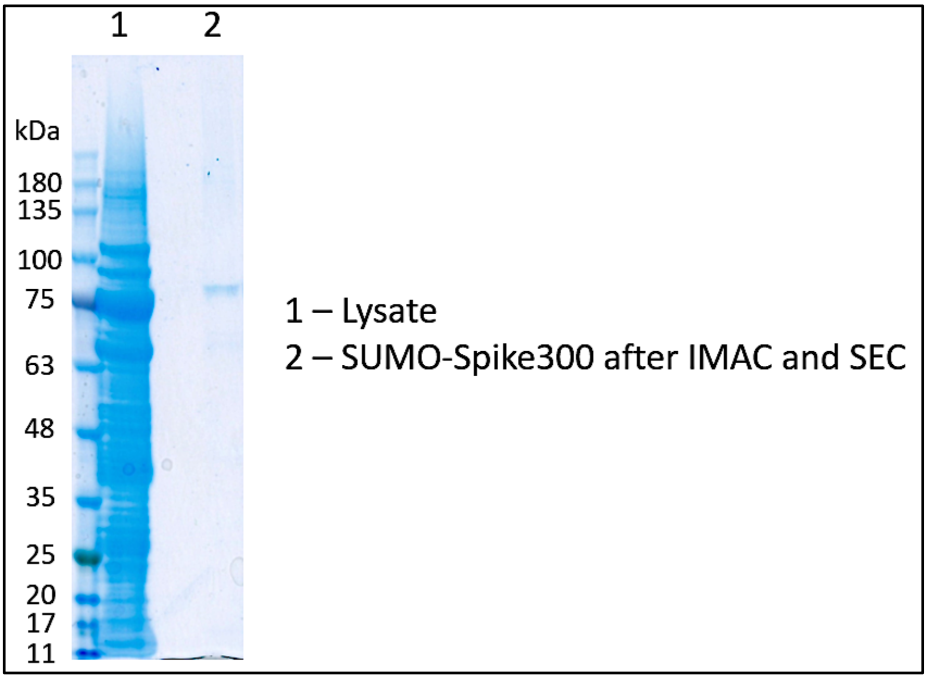

Figure S3. SDS-PAGE gel demonstrating SUMO-Spike300 protein purity after production.

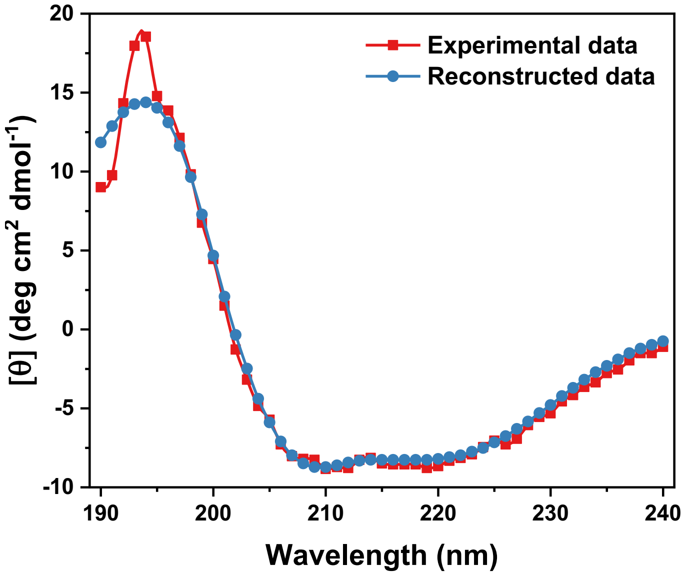

NRMSD:0.128

Result	Helix1	Helix2	Strand1	Strand2	Turns	Unordered	Total
Guess	0.031	0.031	0.307	0.142	0.165	0.323	**0.999**
SVD	0.101	0.065	0.009	0.009	0.046	0.090	**0.32**
Convergent	0.030	0.082	0.274	0.122	0.192	0.276	**0.976**
Stage2	**0.030**	**0.082**	**0.274**	**0.122**	**0.192**	**0.276**	**0.976**
final	**0.030**	**0.086**	**0.266**	**0.123**	**0.201**	**0.287**	**0.993**

Figure S4. Circular dichroism (CD) spectra of the truncated SARS-CoV-2 spike protein (first 300 amino acids). The amount of secondary structure (%) is given in table above.

Saline stability-induced aggregation studies by absorbance.

A solution of NaCl was made up ([NaCl] = 1 M) followed by a 25 μL six-step two-fold serial dilution in a clear, flat bottom, half area 96-well microtitre plate. 25 μL of NeuNAc-$PHEA_{55}$@AuNRs was added to each well and incubated at room temperature for 30 mins. After 30 minutes, an absorbance spectrum was recorded from 400 nm-990 nm with 1 nm intervals.

Spike protein-binding studies by absorbance.

A stock solution of the spike protein (S1) was made up (1.2 $mg.mL^{-1}$) as described in previous section in 10 mM HEPES buffer with 0.15 M NaCl. A 25 μL six-step two-fold serial dilution was made up in the same buffer in a clear, flat bottom, half area 96-well microtitre plate. 25 μL of the NeuNAc-$PHEA_{55}$@AuNRs/ Gal-$PHEA_{55}$@AuNRs/ NeuNAc-$PHEA_{55}$@AuNPs were added to each well and incubated at room temperature for 30 mins. After 30 minutes, an absorbance spectrum was recorded from 400 nm-990 nm with 1 nm intervals. All UV-Vis spectra were normalized to 930 nm glycopolymer coated nanorods and to 400 nm spherical nanoparticles for better comparability. A kinetic study was also conducted by monitoring the absorbance at 508 and 785 nm over 5 hours with 10 mins intervals.

Lectin-binding studies by absorbance.

A stock solution of the lectins SBA and MAL II and SNA were made up (2 $mg.mL^{-1}$) in 10 mM HEPES buffer with 0.15 M NaCl, 0.1 mM $CaCl_2$ and 0.01 mM $MnCl_2$. A 25 μL six-step two-fold serial dilution was made up in the same buffer in a clear, flat bottom, half area 96-well microtitre plate. 25 μL of the NeuNAc-$PHEA_{55}$@AuNRs was added to each well and incubated at room temperature for 30 mins. After 30 minutes, an absorbance spectrum was recorded from 400 nm-990 nm with 1 nm intervals. All UV-Vis spectra were normalized to 930 nm for better comparability.

Lentivirus - binding studies by absorbance.

A 25 μL six-step two-fold serial dilution (1 in 2) of lentivirus (start concentration 2.5 x 10^5 TU/mL) was made in water in a flat bottom, half area, 96-well microtitre plate. 25 μL of the NeuNAc-$PHEA_{55}$@AuNRs was added to each well or 25 μL water to determine the background of lentivirus in the media it was supplied in. The absorbance spectra between 400 and 900 nm in intervals of 10 nm was measured at 5 mins, 25 mins, 45 mins, 65 mins and 90 mins. Each measurement was made in triplicate. All UV-Vis spectra were normalized to 930 nm for better comparability.

Clinical swab samples - binding studies by absorbance.

This study used remnant elutions from nasal, or nasal + oral swab samples collected from symptomatic staff/patients at the University Hospital Coventry and Warwickshire NHS Trust (UHCW) and routinely tested by standard PCR protocols employing the Abbott assay (Ref: 09N77-095,https://www.molecular.abbott/sal/9N77-095_SARS-CoV-2_US_EUA_Amp_PI.pdf) during April-October 2020.[1] As this evaluation study used left-over anonymized material no written informed consent was obtained, although the project was registered with the local COVID-19 research committee.

Dry cotton swabs, one nose and one throat, were obtained in a single universal container. To each primary swab sample was added 2000 μL of molecular grade water (if one swab) or 2500 μL of molecular grade water (if two swabs are in universal container). These were then vortexed and allowed to settle for 5 minutes. All liquid was transferred from primary container into 13 mm × 75 mm tube. These tubes are heat inactivated at 85 °C for 10 minutes. Samples were aliquoted and stored at -80 °C.

For this study four samples were used (Ct 7.74, 14.34, 19.07 and a negative as determined by RT-PCR carried out at UHCW). Each sample was allowed to warm up to room temperature and 25 μL was added to a flat bottom, half area, 96-well microtitre plate. 25 μL of NeuNAc-PHEA$_{55}$@AuNRs or milliQ H_2O (for background absorbance) was added to each sample in duplicate. The absorbance spectra between 400 and 900 nm in intervals of 10 nm was measured at 0 mins, 20 mins, 40 mins, 60 mins. After 60 mins the absorbance spectra measured between 400 and 990 nm in intervals of 1 nm. All UV-Vis spectra were normalized to 930 nm for better comparability.

Supporting Characterization Data for PFP/NeuNac-PHEA$_{55}$

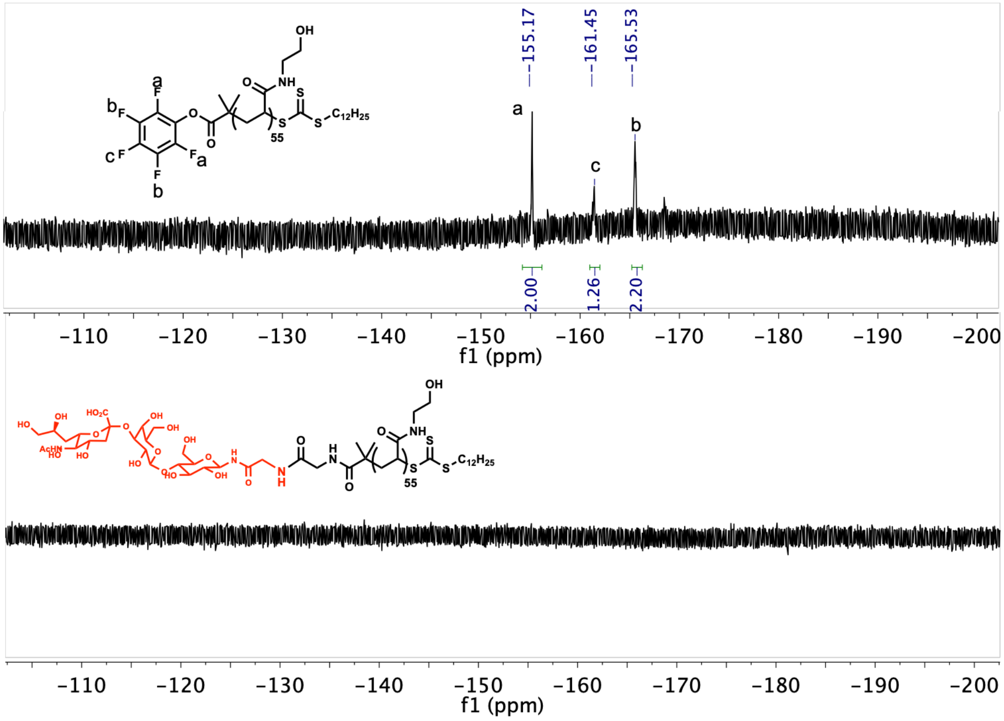

Figure S5. ^{19}F NMR spectra for the purified PFP/NeuNAc-PHEA$_{55}$ homopolymer before and after post-functionalization with 2,3 sialyllactose-glycine. All spectra were recorded in methanol-d_4.

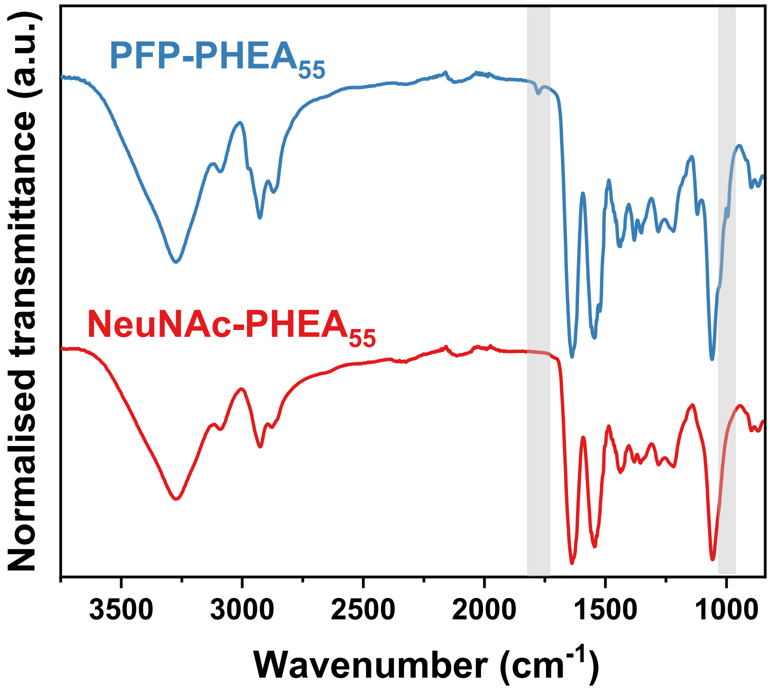

Figure S6. FT-IR spectra for PHEA$_{55}$ homopolymer before (black) and after (red) end-group modification with 2,3 sialyllactose-glycine. The disappearance of the characteristic vibration peaks of PFP group at 950 and 1750 cm^{-1} is shown.

Supporting X-Ray Photoelectron Spectroscopy Data for PFP/NeuNac-PHEA$_{55}$-functionalized AuNRs

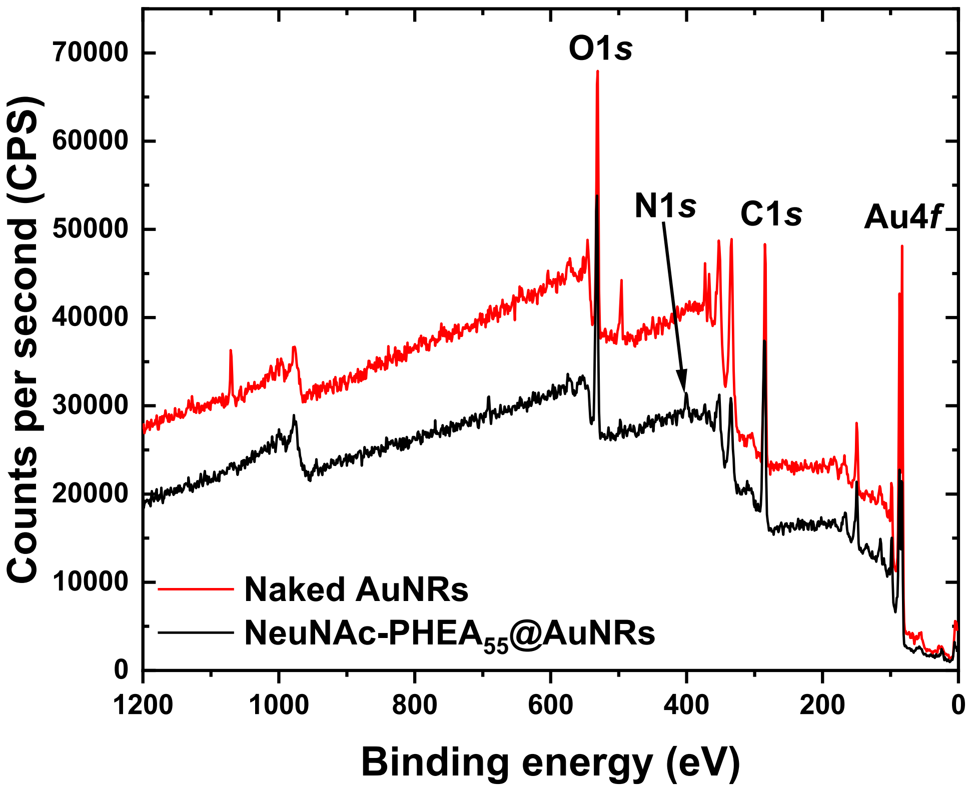

Figure S7. Representative XPS survey scans of citrate-stabilized AuNRs (red) and NeuNAc-PHEA$_{55}$@AuNRs (black).

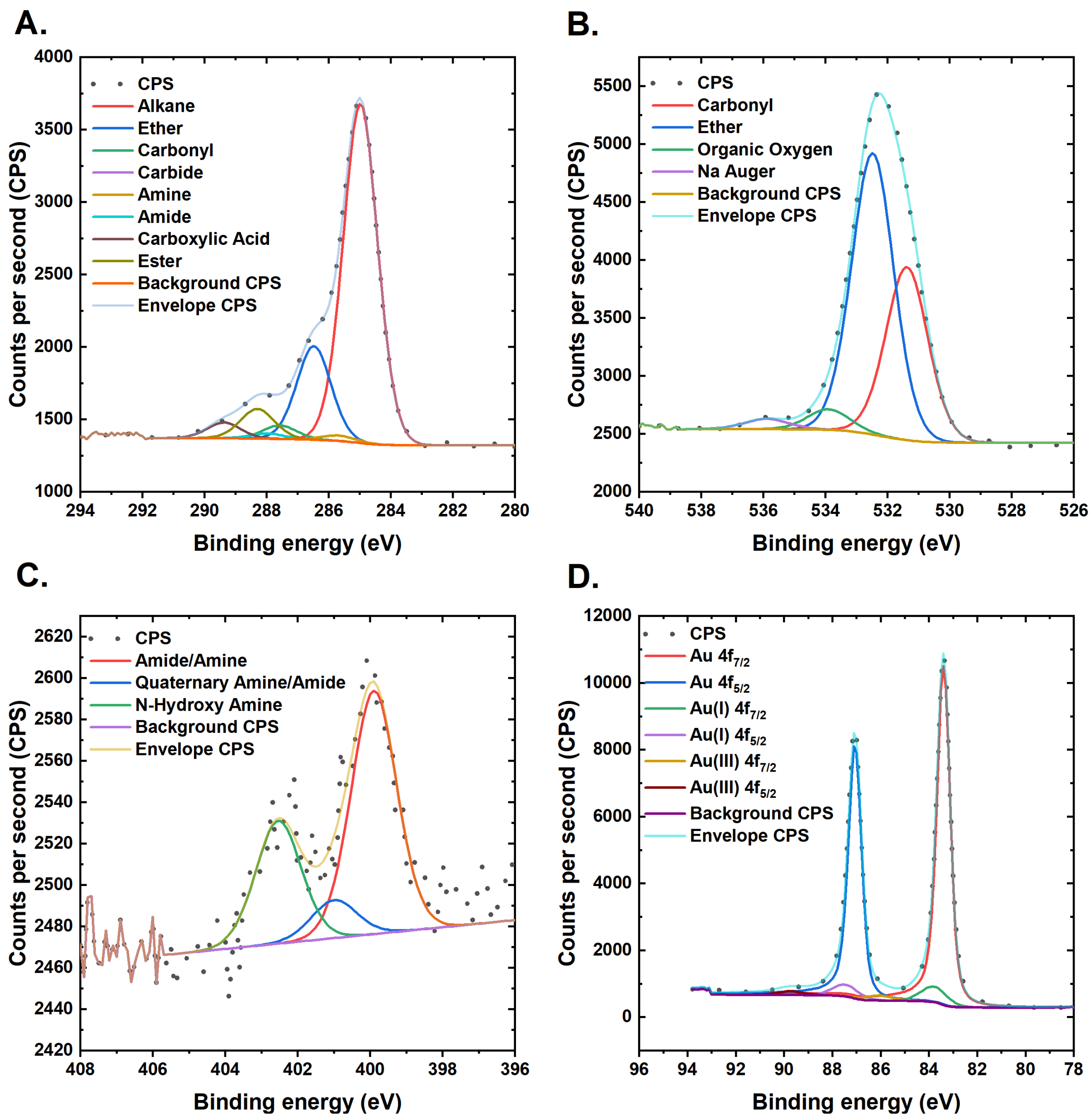

Figure S8. XPS of naked (citrate-stabilized) AuNRs A) C 1*s* B) O 1*s* C) N 1*s* and D) Au 4*f*.

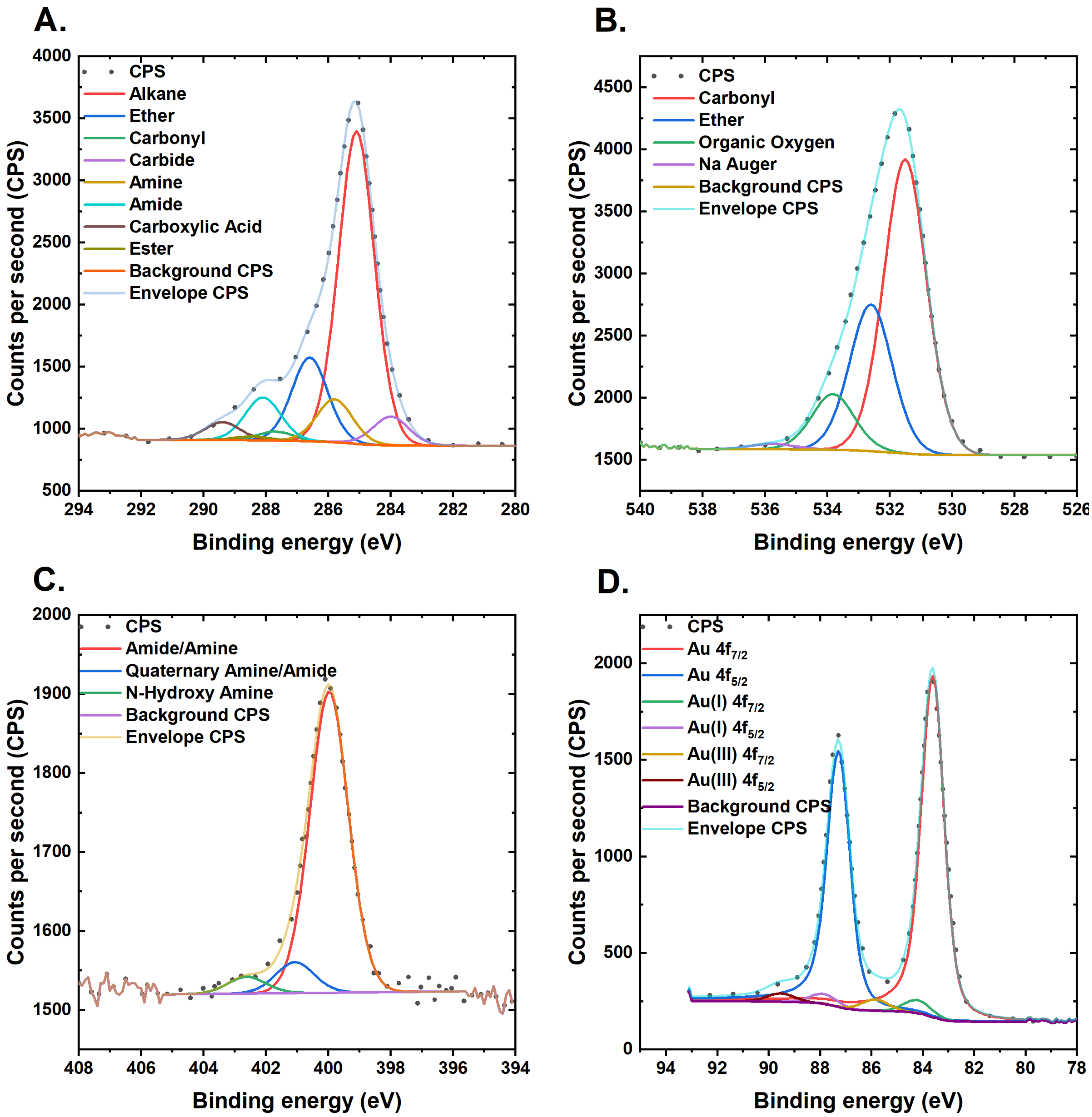

Figure S9. XPS of PFP-PHEA$_{55}$@AuNRs A) C 1*s* B) O 1*s* C) N 1*s* and D) Au 4*f*.

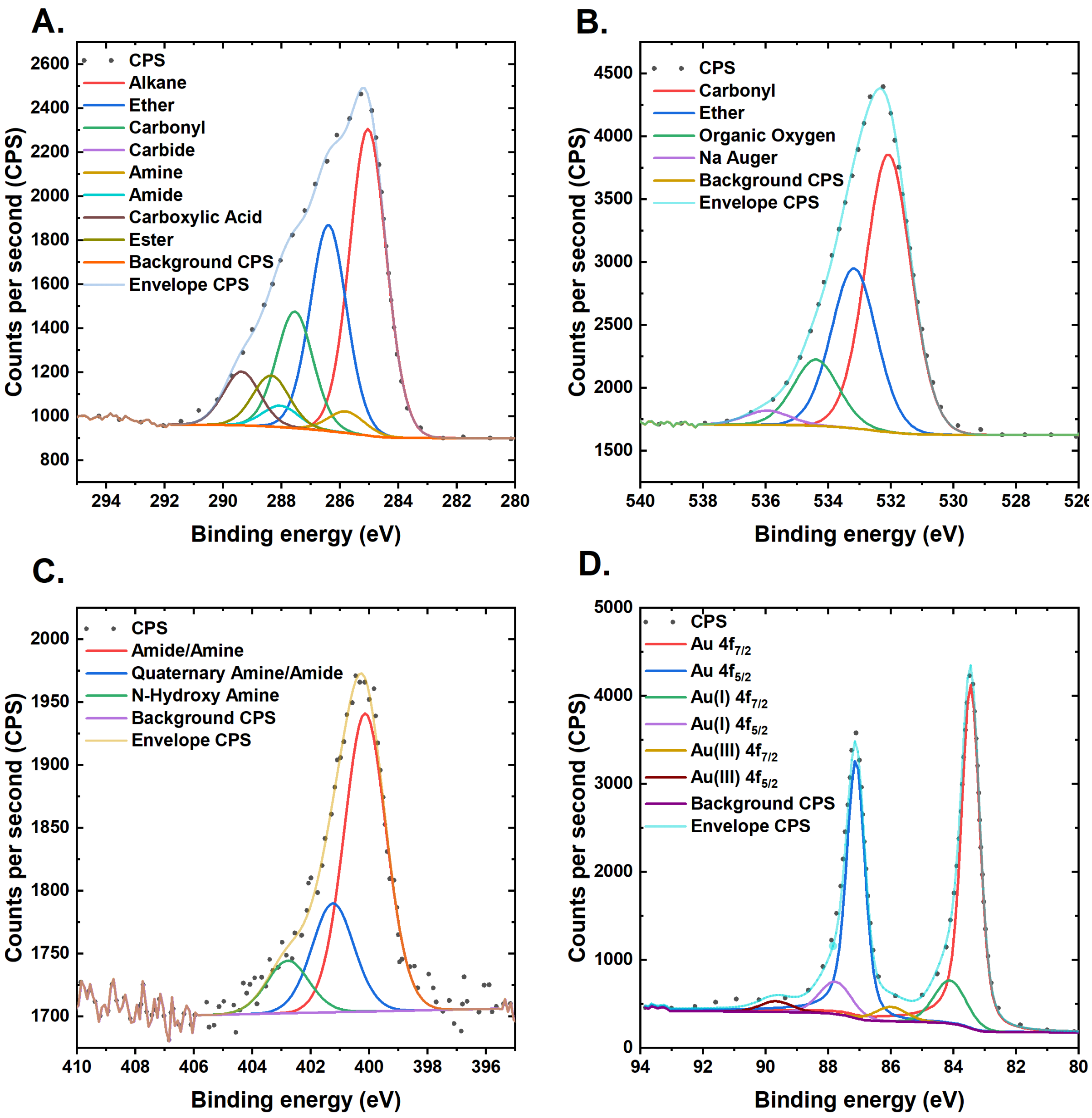

Figure S10. XPS of NeuNAc-PHEA$_{55}$@AuRod A) C 1*s* B) O 1*s* C) N 1*s* and D) Au 4*f*.

Table S1. Elemental compositions of NeuNAc/PFP-PHEA$_{55}$-functionalized gold nanorods determined by XPS

Particle Composition	Elemental Percentage Composition (%)				Elemental Ratios	
	C 1*s*	O 1*s*	N 1*s*	Au 4*f*	N 1*s*/C 1*s*	N 1*s*/Au 4*f*
**Citrate-stabilized AuNRs**	55.96	31.76	2.34	9.95	0.042	0.24
**PFP-PHEA$_{55}$@AuNRs**	65.62	27.67	4.34	2.38	0.066	1.82
**NeuNAc-PHEA$_{55}$@AuNRs**	59.97	31.32	4.38	4.32	0.073	1.01

Table S2. C 1*s* bonding composition of NeuNAc/PFP-PHEA$_{55}$-functionalized gold nanorods determined by XPS

Particle Composition	C 1*s* Bond Percentage Composition (%)								Bond Ratios	
	Alkane	Ether	Carbonyl	Carbide	Amine	Amide	Carboxylic Acid	Ester	Amide/Alkane	Amide/Ether
**Citrate-stabilized AuNRs**	67.41	18.67	2.71	0	1.07	1.07	3.15	5.92	0.016	0.057
**PFP-PHEA$_{55}$@AuNRs**	57.55	15.46	1.69	5.34	7.95	7.96	3.31	0.74	0.138	0.515
**NeuNAc-PHEA$_{55}$@AuNRs**	39.43	26.49	15.01	0	2.76	2.76	6.95	6.6	0.070	0.104

Supporting Characterization Data for Colloidal Stability and Spike protein/Lectin-Binding Studies of NeuNAc-PHEA$_{55}$-Functionalized AuNRs

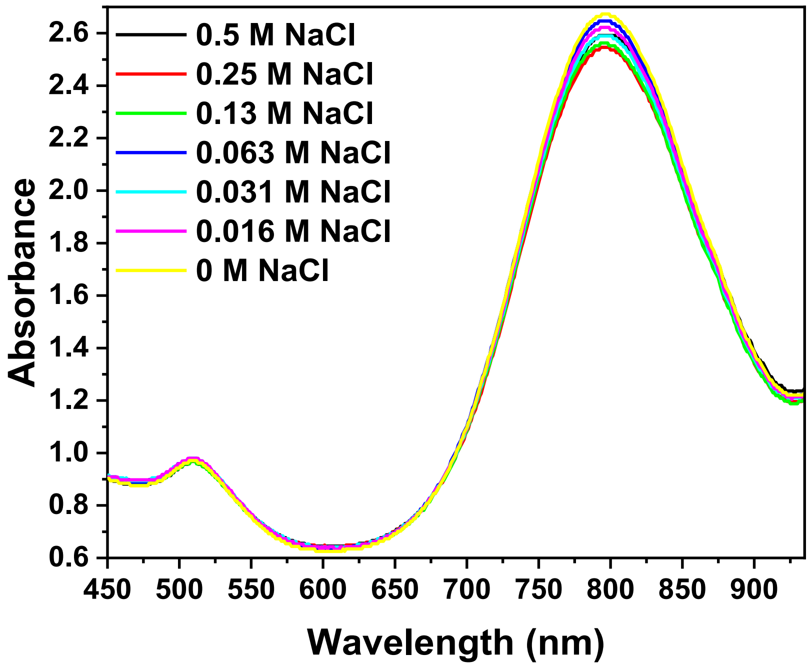

Figure S11. UV-Vis spectra of NeuNAc-PHEA$_{55}$-decorated gold nanorods in response to different concentrations of NaCl.

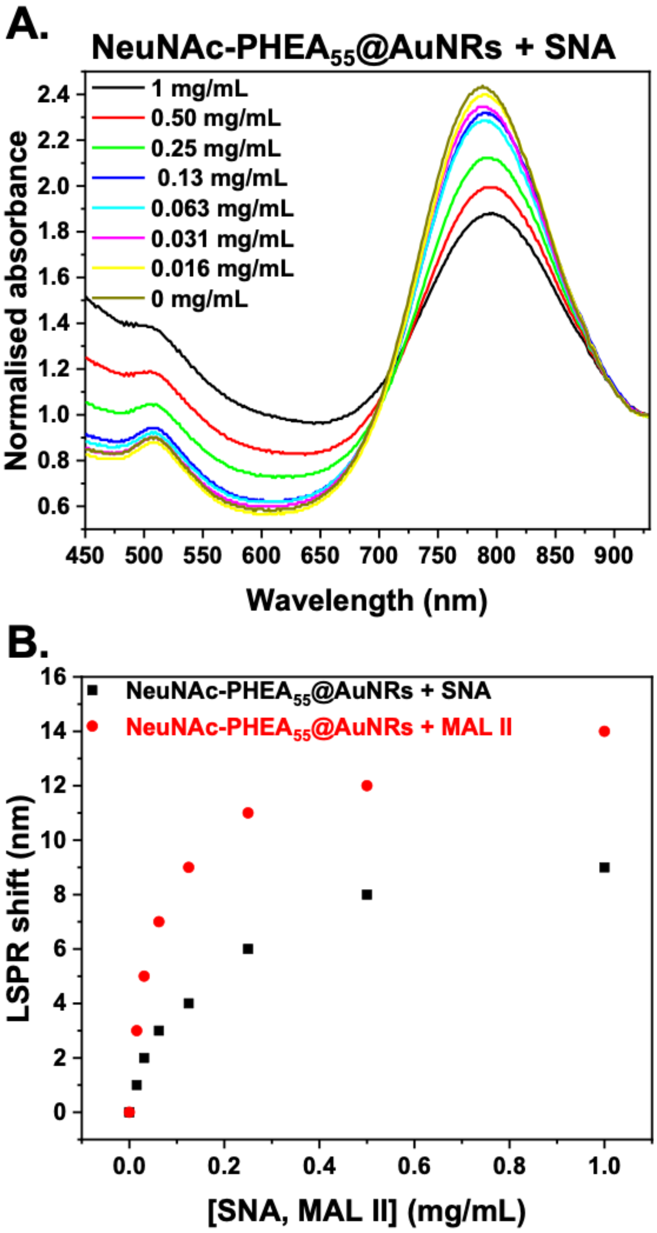

Figure S12. (A) UV- Vis spectra of NeuNAc-PHEA$_{55}$@AuNRs in response to *Sambucus nigra* agglutinin (SNA) lectin and (B) LSPR peak shift of NeuNAc-PHEA$_{55}$@AuNRs as a function of SNA and MAL II concentration, determined by UV-Vis spectroscopy in HEPES buffer.

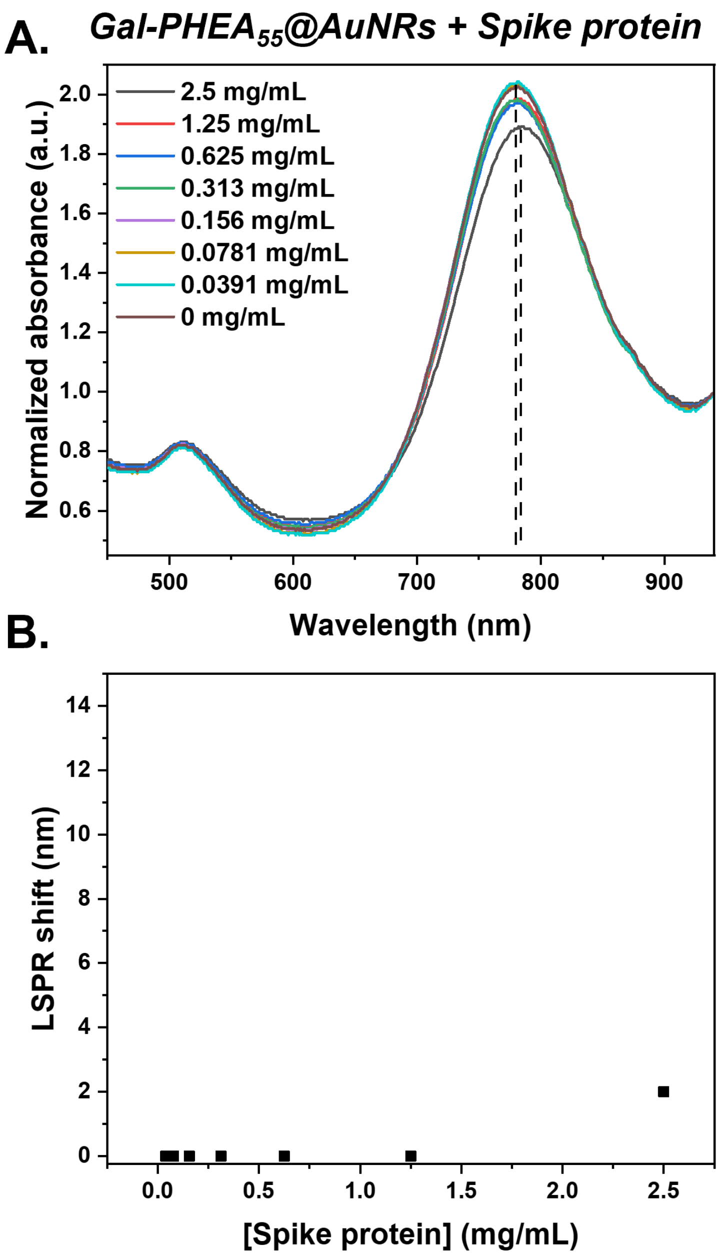

Figure S13. (A) UV- Vis spectra of Gal-PHEA$_{55}$@AuNRs in response to spike protein and (B) LSPR peak shift of Gal-PHEA$_{55}$@AuNRs as a function of spike protein concentration, determined by UV-Vis spectroscopy in HEPES buffer.

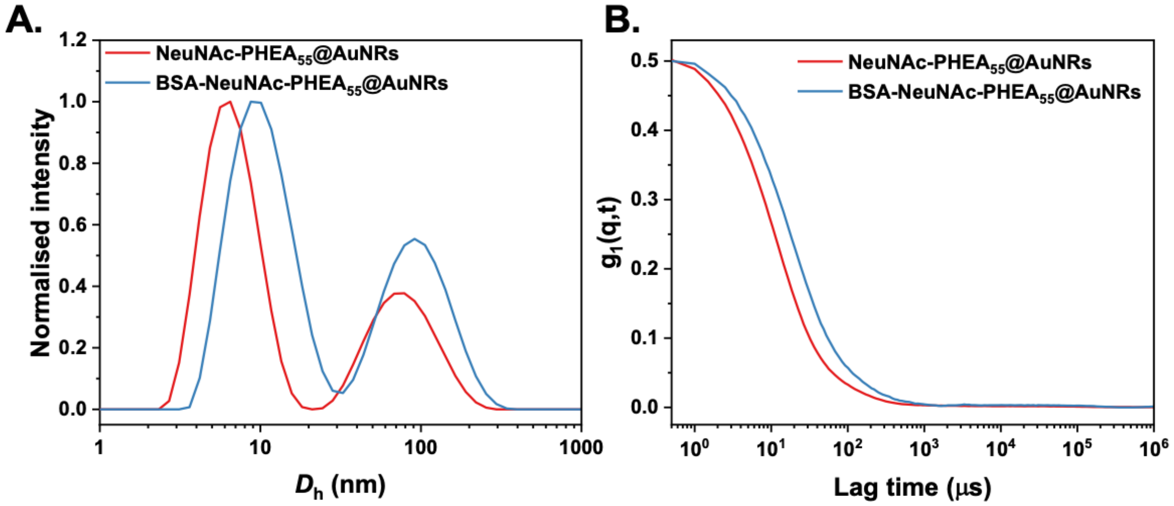

Figure S14. (A) Intensity-weighted size distributions and corresponding autocorrelation functions (B) for NeuNAc-PHEA$_{55}$@AuNRs before (red curves) and after (blue curves) incubation with BSA.

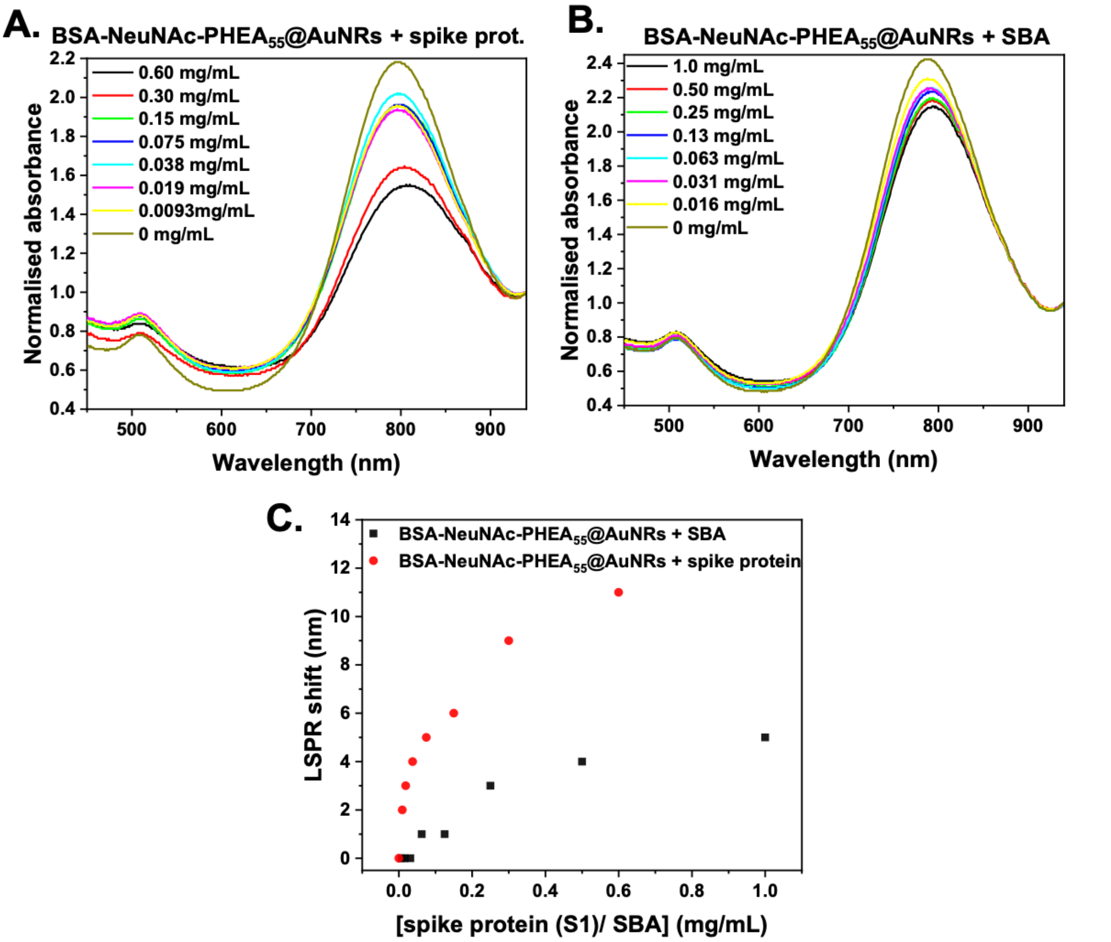

Figure S15. UV-Vis spectra of BSA-coated NeuNAc-PHEA$_{55}$@AuNRs in response to spike protein (A) and SBA lectin (B). LSPR peak shift of BSA-NeuNAc-PHEA$_{55}$@AuNRs as a function of spike protein and SBA concentration, determined by UV-Vis spectroscopy in HEPES buffer.

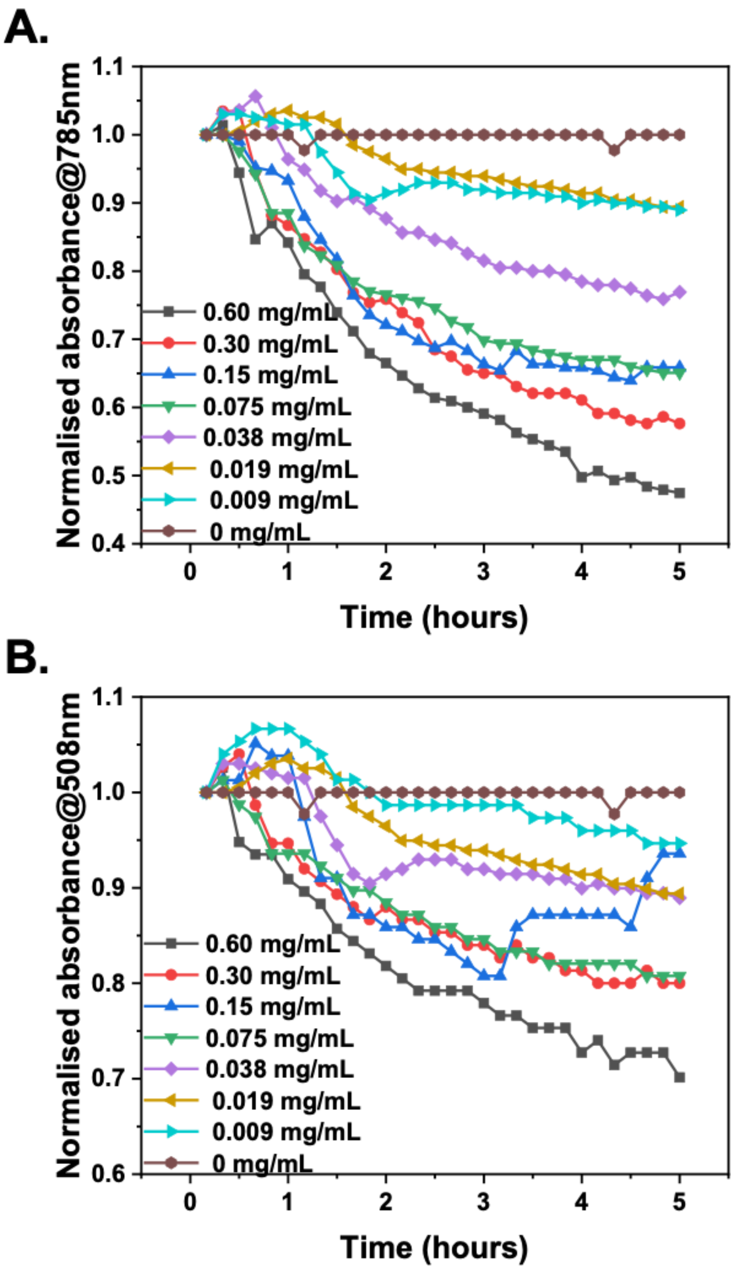

Figure S16. Kinetics of NeuNAc-PHEA$_{55}$@AuNRs in response to different concentrations of spike protein (0.6-0.01 mg/mL) by monitoring absorbance@785 (A) and 508 (B) over 5 hours with 10 minutes intervals.

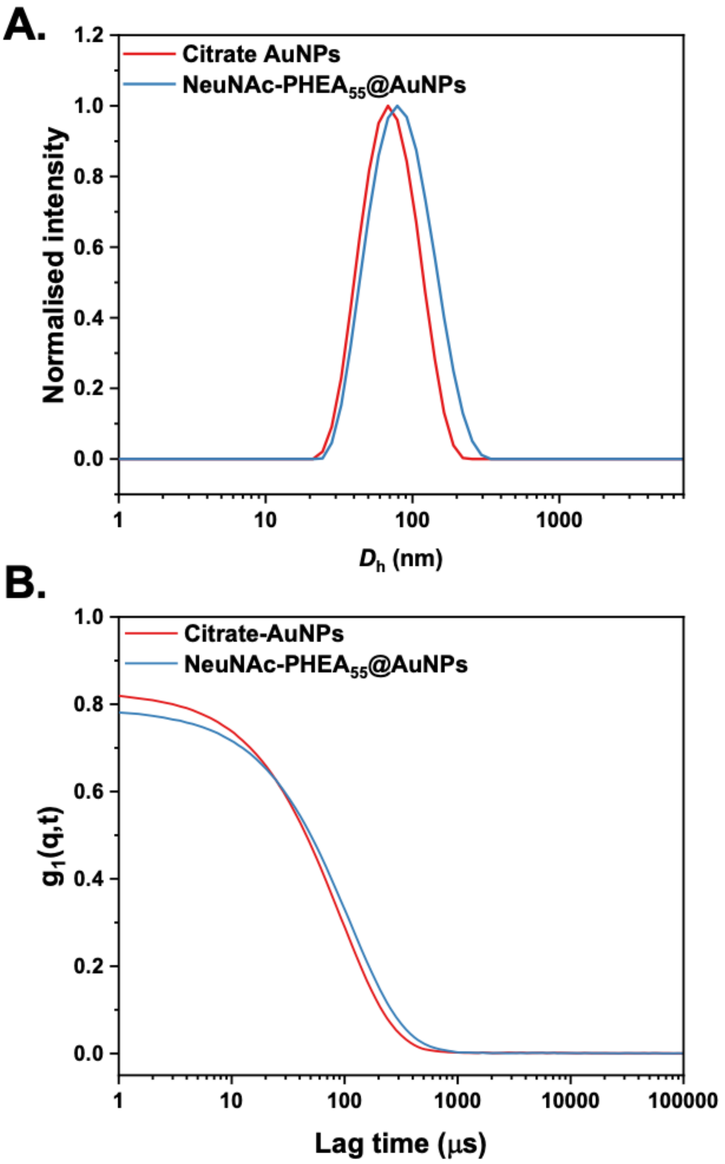

Figure S17. (A) Intensity-weighted size distributions and corresponding autocorrelation functions (B) before (red curve) and after (blue curve) coating of citrate spherical AuNPs (40 nm) with NeuNAc-PHEA$_{55}$.

Supporting Characterization Data of NeuNAc-PHEA$_{55}$-Functionalized AuNRs in Response to Lentiviral and Clinical Swab Samples

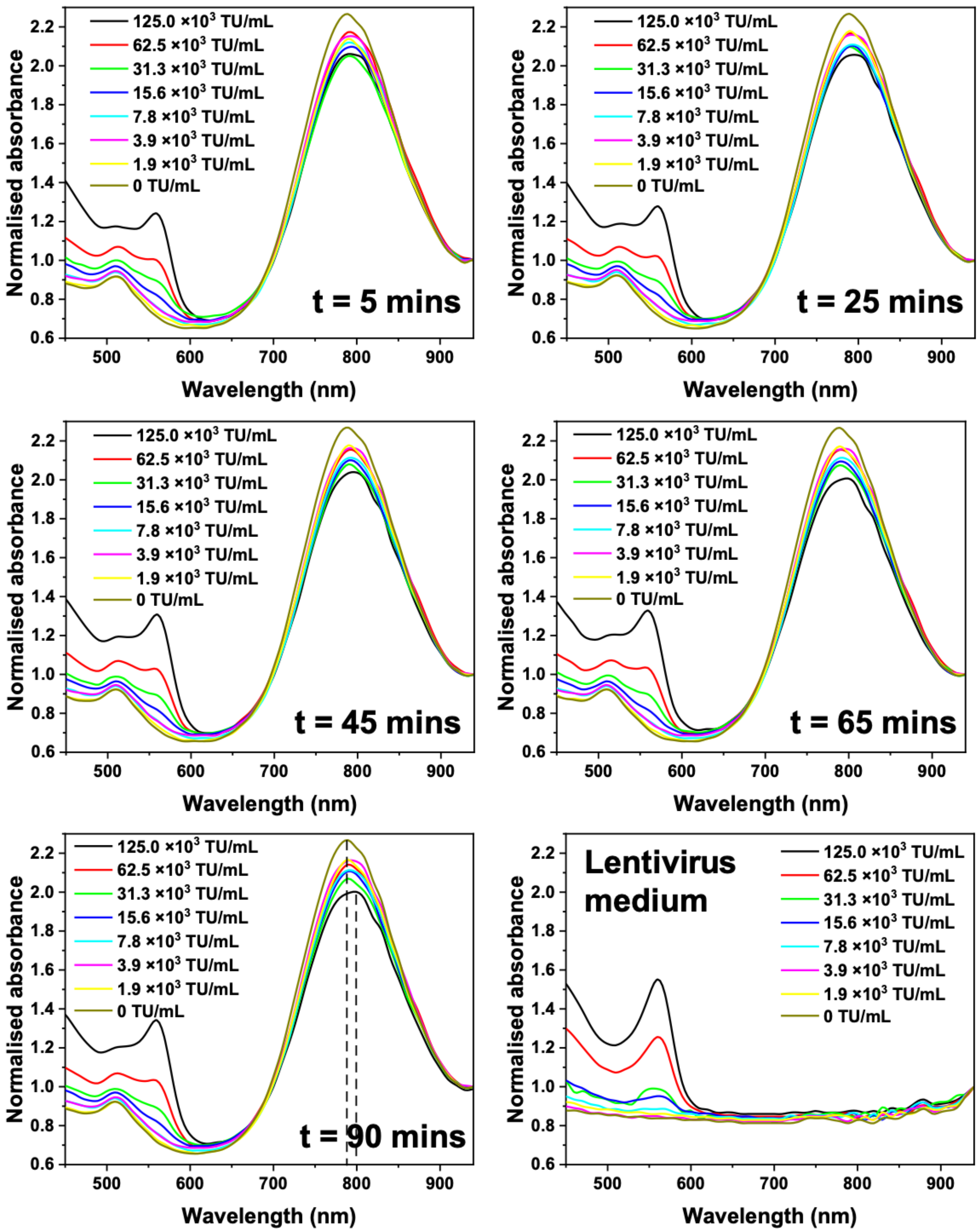

Figure S18. UV-Vis spectra of NeuNAc-PHEA$_{55}$@AuNRs in response to Spike (SARS-CoV-2) pseudotyped lentivirus at t = 5, 25, 45, 65 and 90 mins. UV-Vis spectra of lentiviral media are also shown.

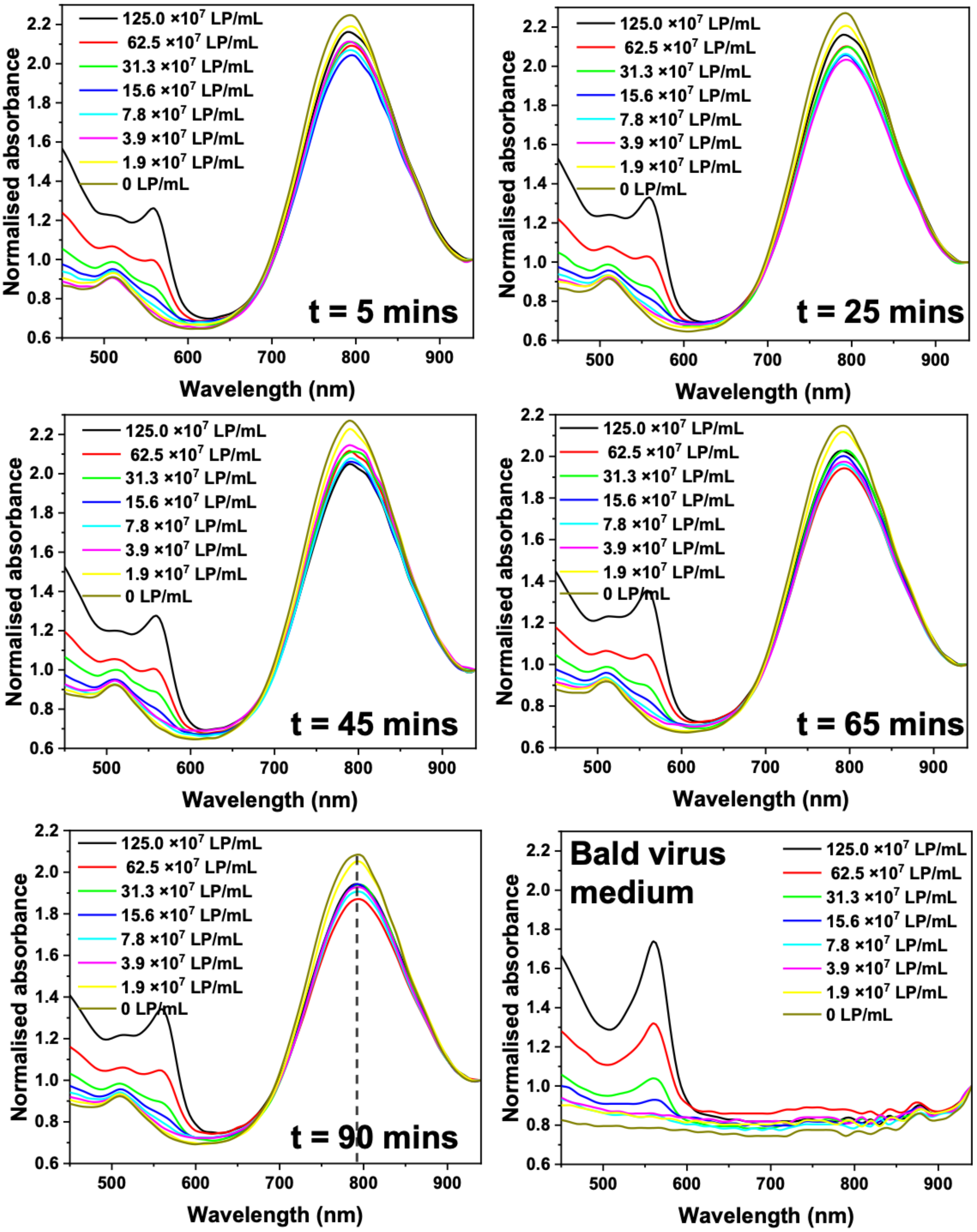

Figure S19. UV-Vis spectra of NeuNAc-PHEA$_{55}$@AuNRs in response to Spike (SARS-CoV-2) bald pseudotyped lentivirus at t = 5, 25, 45, 65 and 90 mins. UV-Vis spectra of lentiviral media are also shown.

The lentiviral data includes spike positive (S17) and spike negative (S18) lentiviral samples. It is crucial to note the concentration units are not identical. The positives is in TU/mL, which is transduction units/mL determined by a cell based assay for integration into a cell. However,

the bald do not integrate into the cell (as they are missing the spike protein which is being tested for in the positive assay) meaning a total particles/mL value is used, which is estimated. Hence comparison between them is challenging in the assay used here, which shows a continuum response. In both case there was a decrease in absorbance upon addition of increasing concentrations of lentivirus. The positive sample did show a shoulder forming plus a shift to longer wavelength (suggesting binding was occurring) but the data show was collected at a resolution of 10nm, and hence we do not want to claim a significant change. This data is included here for completeness to aid future studies. The spike protein and clinical sample studies do show a clear binding response.

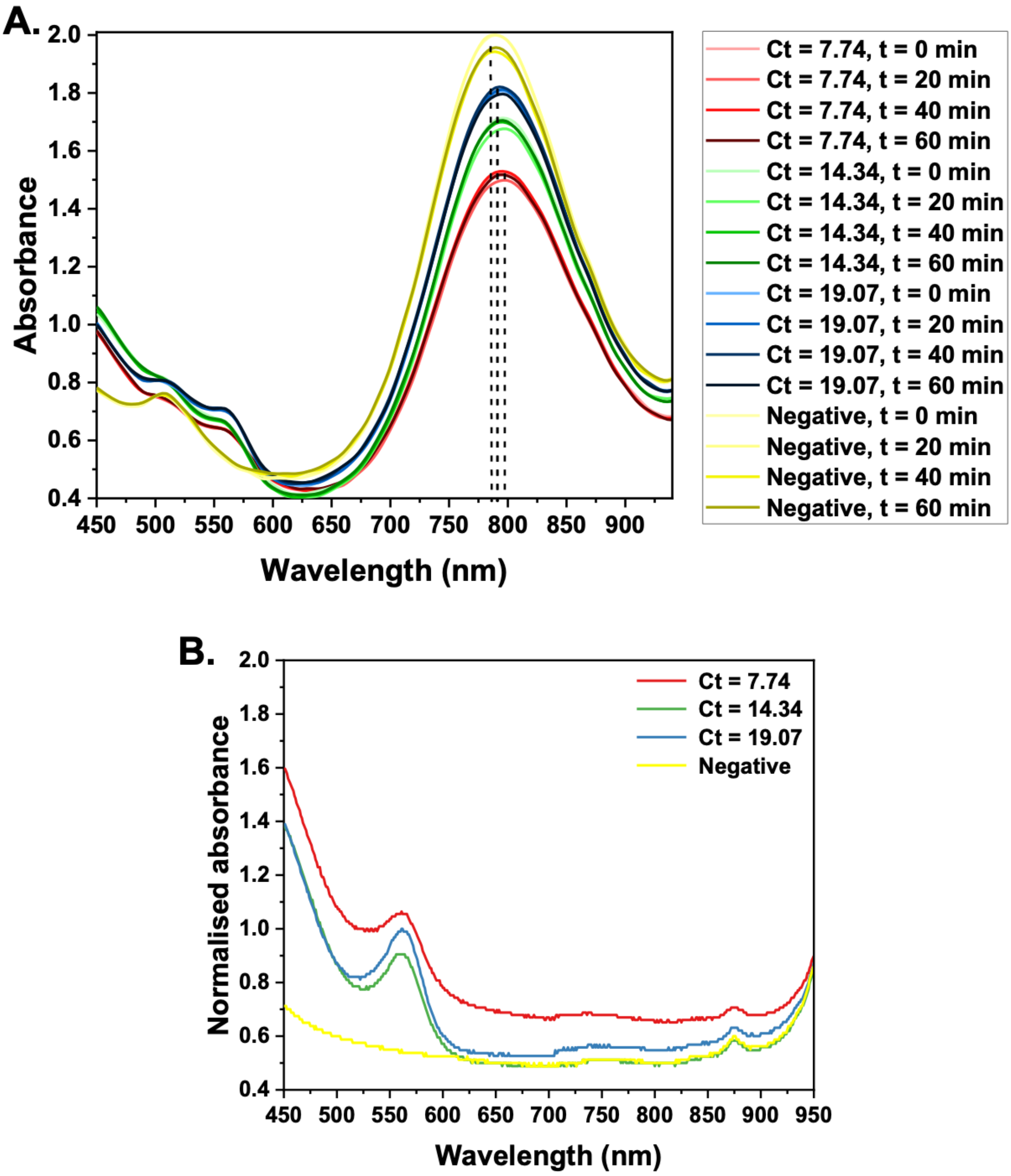

Figure S20. UV-Vis spectra of NeuNAc-PHEA$_{55}$@AuNRs in response to clinical swab samples with different viral loads (Ct = 7.74, 14.34, 19.07) at t = 0, 20, 40 and 60 mins (A). UV-Vis spectra of swab samples media are also shown (B).

References

1. Abbott Molecular Inc. *Abbot Real Time SARS-CoV-2 Instructions for Use (Ref: 09N77 095);* 2020.
